# Cecal Ligation and Puncture-Induced Sepsis Promotes Brown Adipose Tissue Inflammation Without Any Impact on Expression of Thermogenic-Related Genes

**DOI:** 10.3389/fphys.2021.692618

**Published:** 2021-07-12

**Authors:** José María Moreno-Navarrete, Ferran Comas, Vincent de Jager, José Manuel Fernández-Real, Hjalmar R. Bouma

**Affiliations:** ^1^Department of Diabetes, Endocrinology and Nutrition (UDEN), Hospital of Girona “Dr Josep Trueta” and Institut d’Investigació Biomèdica de Girona (IdIBGi), Girona, Spain; ^2^CIBER de la Fisiopatología de la Obesidad y Nutrición (CIBERobn) (CB06/03/010), Girona, Spain; ^3^Department of Medicine, Universitat de Girona, Girona, Spain; ^4^Department of Clinical Pharmacy and Pharmacology, University Medical Center Groningen, University of Groningen, Groningen, Netherlands; ^5^Department of Internal Medicine, University Medical Center Groningen, University of Groningen, Groningen, Netherlands

**Keywords:** cecal ligation and puncture, inflammation, sepsis, brown adipose tissue, white adipose tissue

## Abstract

**Background and Aims:** The negative effects of chronic low-level inflammation on adipose tissue physiology have been extensively demonstrated, whereas the effects of acute inflammation are less studied. Here, we aimed to investigate the effects of sepsis-induced acute inflammation on gene expression markers of brown and white adipose tissue functionality.

**Methods:** Brown adipose tissue (BAT) and perirenal white adipose tissue (prWAT) gene expression markers were analyzed in cecal ligation and puncture (CLP)-induced sepsis mice model.

**Results:** CLP-induced sepsis attenuated expression of adipogenesis-related genes, in parallel to increased *Tnf, Il6*, and *Ltf* gene expression in prWAT. In contrast, CLP-induced sepsis resulted in increased expression of pro-inflammatory genes (*Il6, Ltf*, and *Lbp*) in BAT, without affecting expression of genes encoding for thermogenic activity.

**Conclusion:** Sepsis promotes both prWAT and BAT inflammation, associated with reduced adipogenesis-related gene expression in prWAT, without significant effects on BAT thermogenic genes.

## Introduction

Obesity is a low-level chronic inflammatory condition associated with adipose tissue dysfunction and insulin resistance mediated by decreased levels of adiponectin and increased proinflammatory cytokines (Klöting and Blüher, 2014). Adipose tissue dysfunction is characterized by increased inflammation and decreased adipogenesis (Klöting and Blüher, 2014; Carobbio et al., 2017). While adipose tissue dysfunction induced by chronic inflammation is well demonstrated (Klöting and Blüher, 2014; Acosta et al., 2016; Carobbio et al., 2017; Hotamisligil, 2017), the impact of acute inflammation on adipose tissue is not yet fully understood, in particular brown adipose tissue (BAT).

Acute inflammation induced by bariatric surgery results in increased expression of pro-inflammatory genes in parallel to decreased adipogenesis-related gene expression in both human subcutaneous and visceral fat depots (Ortega et al., 2016). Exposure to bacterial endotoxin lipopolysaccharide (LPS) leads to adipocyte and white adipose tissue (WAT) dysfunction, promoting adipocyte inflammation, decreasing adipogenesis, and inhibiting insulin-induced glucose uptake (Creely et al., 2007; Lu et al., 2008; Osto et al., 2011; Guo et al., 2015).

Cecal ligation and puncture (CLP)-induced sepsis in obese mice results in increased expression of pro-inflammatory cytokines (TNF-α and IL-6) in visceral WAT (Tsujimura et al., 2011; Kutsukake et al., 2014) and decreased circulating adiponectin levels (Kaplan et al., 2016). Adiponectin is the main peptide produced by adipocytes and is involved in an array of metabolic processes, including insulin sensitivity, inflammation, and angiogenesis (Xia et al., 2018). Acute depletion of adiponectin aggravates insulin resistance and hyperlipidemia, as compared to mice with congenital loss of adiponectin (Xia et al., 2018). Hence, decreased adiponectin levels might underlie the insulin resistance that is observed in inflammatory states. Thus, acute inflammation is associated with WAT dysfunction, resulting in a significant reduction in adiponectin levels and insulin resistance, similar as observed in chronic inflammation.

Despite these previous findings in about the negative effect of CLP on WAT, increasing expression of proinflammatory cytokines but decreasing adiponectin (Tsujimura et al., 2011; Kutsukake et al., 2014), to our knowledge, the acute effects of sepsis on metabolic-related pathways in WAT and BAT have not been investigated. In WAT, the main metabolic-related pathways are composed of genes involved in fatty acid and glucose intake (*Fabp4 and Slc2a4*), adipogenesis (*Adipoq and Pparg*), lipogenesis (*Fasn, Acaca, and Scd1*), lipid droplet development (*Plin1*), and lipolysis (*Prkaca, Mgll, and Lipe*) (Klöting and Blüher, 2014; Carobbio et al., 2017). In BAT, the most important metabolic-related genes include those involved thermogenesis (*Ucp1, Prdm16, and Dio2*), mitochondrial biogenesis and activity (*Pgc1a and Cycs*), fatty acid and glucose uptake, and lipolysis (Wang et al., 2021). Even though UCP2 does not have the uncoupling potential of UCP1 (Nicholls, 2021), this protein is required to BAT thermogenesis and to the adaptation to cold exposure, by promoting the utilization of non-esterified fatty acids (Nakae et al., 2008; Caron et al., 2017). Full name of these genes is detailed in Table 1.

**Table 1:** Description of TaqMan primer/probe sets used in gene expression analysis.

Gene full name	Gene symbol	TaqMan assay reference
Interleukin 6	*Il6*	Mm00446190_m1
Tumor necrosis factor	*Tnf*	Mm00443258_m1
Lipopolysaccharide-binding protein	*Lbp*	Mm00493139_m1
Lactoferrin	*Ltf*	Mm00434787_m1
Uncoupling protein 1 (mitochondrial, proton carrier)	*Ucp1*	Mm01244861_m1
PR domain containing 16	*Prdm16*	Mm00712556_m1
Cytochrome c, somatic	*Cycs*	Mm01621048_s1
Uncoupling protein 2 (mitochondrial, proton carrier)	*Ucp2*	Mm00627599_m1
Deiodinase, iodothyronine, and type II	*Dio2*	Mm00515664_m1
Peroxisome proliferative-activated receptor, gamma, and coactivator 1 alpha	*Pgc1a*	Mm01208835_m1
Aconitase 1	*Aco1*	Mm00801417_m1
Protein kinase, cAMP-dependent, catalytic, and alpha	*Prkaca*	Mm00660092_m1
Lipase, hormone sensitive	*Lipe*	Mm00495359_m1
Monoglyceride lipase	*Mgll*	Mm00449274_m1
Fatty acid synthase	*Fasn*	Mm00662319_m1
Stearoyl-coenzyme A desaturase 1	*Scd1*	Mm00772290_m1
Perilipin 1	*Plin1*	Mm00558672_m1
Leptin	*Lep*	Mm00434759_m1
Peroxisome proliferator-activated receptor gamma	*Pparg*	Mm00440940_m1
Adiponectin	*Adipoq*	Mm00456425_m1
Fatty acid-binding protein 4, adipocyte	*Fabp4*	Mm00445880_m1
Solute carrier family 2 (facilitated glucose transporter), member 4	*Slc2a4* or *Glut4*	Mm01245502_m1
Acetyl-coenzyme A carboxylase alpha	*Acaca*	Mm01304258_m1

In both WAT and BAT, the good performance of metabolic-related pathways contributes to whole-body metabolic homeostasis, by ensuring proper lipid storage (WAT) and enhancing energy expenditure and attenuating fat mass gain (BAT) (Klöting and Blüher, 2014; Carobbio et al., 2017; Wang et al., 2021).

Here, we hypothesized that acute inflammation associated with CLP-induced sepsis might impact on gene expression markers of metabolic-related pathways in WAT and BAT. To test this hypothesis, in the present study, we aimed to investigate gene expression markers of adipogenesis, lipogenesis, lipid droplet development, fatty acid and glucose uptake, and lipolysis in perirenal white adipose tissue (prWAT) and markers of thermogenesis, mitochondrial biogenesis and activity in BAT in CLP-induced sepsis mouse experimental model.

## Materials and Methods

### Mice Experiment

#### Animals

Adult male C57/Bl6j mice (8–12 weeks old, Charles River, the Netherlands) were housed at a light:dark cycle of 12 h:12 h. Animals were fed *ad libitum* using standard animal lab chow and drinking water. Experiments were approved by the Central Committee Animal Experiments of the Netherlands (protocol number 16593).

#### Animal Experiment

Animals were anesthetized by subcutaneous injection of xylazine/ketamine (100/10 mg/kg), followed by administration of buprenorphine (0.1 mg/kg) as analgesic. After confirmation of anesthesia by lack of response to paw pinch and eye reflex, the abdomen was shaved, cleaned, and degermed using a povidone-iodine solution before a 1-cm midline incision was made. The cecum was ligated with a 6–0 suture at half the distance between distal pole and the base of the cecum and punctured once with a 21-gauge needle (‘through-and-through’ from mesenteric toward anti-mesenteric direction) which is expected to lead to ‘mid-grade’ sepsis (Newcomb et al., 1998; Turnbull et al., 2004; Rittirsch et al., 2009). A small amount of stool (2–3 mm) was then extruded to ensure wound patency. The cecum was repositioned, thereby taking care not to spill fecal material on the wound edges, followed by closure of the abdomen by running sutures to the abdominal musculature (6–0 Safil sutures) and short interrupted sutures to the skin (5–0 Safil), as described by Rittirsch et al., 2009. Next, 1 ml of saline (warmed, 0.9% NaCl s.c.) was administered to compensate for the expected relative volume depletion due to the onset of sepsis. Following the procedure, mice recovered at 26–28°C for 2 h. Broad-spectrum antibiotics (imipenem/cilastatin, 100 mg/kg s.c.) were administered at 2–10 h following surgery, while analgesics (buprenorphine, 0.1 mg/kg body weight, s.c.) were administered perioperatively. A group of operated animals, in which the cecum was located but not punctured, served as sham. In addition, a group of animals were included that underwent time-matched anesthesia, but no surgery and served as controls. Animals were euthanized at 24 h after surgery. Upon euthanization, 40 μl of blood was used to obtain a blood cell counts on a Sysmex PoCH 100-iv analyzer hematocytometer. In addition, a blood smear was Giemsa-stained to confirm the data from the hematocytometer and manually obtain differential blood cell counts. The remainder of the EDTA-anticoagulated blood was separated into plasma by centrifugation at 1,600 *g* for 10 min and serum by allowing it to clot for 30 min followed by centrifugation at 3,000 g for 10 min. Plasma, serum, and interscapular BAT and prWAT were snap-frozen in liquid nitrogen for further analysis.

#### Measurement of Cytokines

To determine the extent of inflammation and assess the severity of sepsis, plasma levels of TNF-α and IL-6 were measured, in addition to changes in body weight, temperature, and leukocyte counts. Mouse DuoSet ELISAs were used to measure plasma cytokine levels (DY410 and DY406; RnD Systems), according to the manufacturer’s instructions. Briefly, ELISA plates (DY990, RnD Systems) were coated overnight with the capture antibody diluted in 100 μl PBS. Plates were washed three times with wash buffer (0.05% Tween^®^ 20 in PBS; 137 mM NaCl, 2.7 mM KCl, 8.1 mM Na_2_HPO_4_, 1.5 mM KH_2_PO_4_, pH 7.2–7.4), followed by blocking by incubating the wells for 1 h with 300 μl of reagent diluent (1% probumin w/v in PBS). Washing was repeated, and samples were added to the wells. Plasma samples were diluted 10x for TNF-α, and 100x for IL-6 in 100 μl reagent diluent, to obtain an OD value within the measurement range as described by the manufacturer. After incubation for 2 h at room temperature, wells were washed, followed by adding 100 μl of detection antibody diluted in reagent diluent to each well. Again, plates were left to incubate for 2 h at room temperature, followed by washing. Finally, 100 μl of substrate solution (DY999, RnD Systems) was added, and after 20 min incubating in the dark, 50 μl stop solution (2 M H_2_SO_4_) was added. The optical density (OD) was measured using a microplate reader set to 450 nm, while readings at 540 nm were subtracted as correction to increase accuracy.

#### RNA Analysis

RNA purification was performed using the RNeasy Lipid Tissue Mini Kit (QIAgen, Izasa SA, Barcelona, Spain), and the integrity was checked with the Agilent Bioanalyzer. Gene expression was assessed by real-time polymerase chain reaction (PCR) using a LightCycler 480 Real-Time PCR System (Roche Diagnostics SL, Barcelona, Spain), with TaqMan technology suitable for relative genetic expression quantification. The real-time PCR was performed in a final volume of 12 μl. The cycle program consisted of an initial denaturing for 10 min at 95°C, 40 consecutive 15-min cycles of denaturizing at 95°C, and a 1-min annealing and extension phase at 60°C. A threshold cycle (Ct value) was obtained for each amplification curve, and a DeltaCt value was first calculated by subtracting the Ct value eukaryotic 18S rRNA endogenous control RNA from the Ct value for each sample. Fold changes compared with the endogenous control were then determined by calculating 2-DeltaCt, so that gene expression results are expressed as expression ratio relative to eukaryotic 18S gene expression according to the manufacturer’s guidelines.

The primer/probe sets used are detailed in Table 1.

#### Statistical Analysis

Statistical analyses were performed using SPSS 12.0 software for Windows (IBM Inc., Armonk, New York, United States) and analyzed using nonparametric tests (Mann–Whitney *U* test). Data are expressed as mean ± standard error of the mean. *p* < 0.05 was considered statistically significant different. Figures were made with GraphPad Prism version 5.00 for Windows (GraphPad Software, San Diego, United States).

## Results

**Figure 1:**
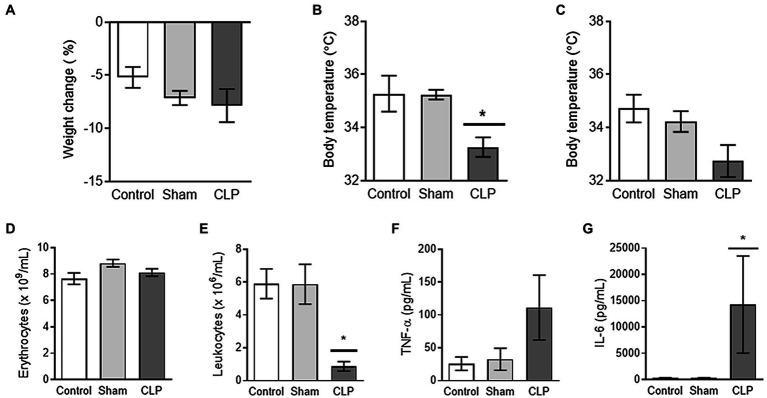
**(A–C)** Sepsis leads to transient hypothermia. Mice in cecal ligation and puncture (CLP) seem to experience weight loss, which was, however, not different compared to control or sham **(A)**. The body temperature after induction of sepsis CLP was transiently reduced at 8 h after induction as compared to control and Sham **(B)**. Upon euthanization (24 h after induction), no differences were observed in body temperatures **(C)**. **(D,E)** Sepsis is associated with profound leukopenia. The numbers of erythrocytes **(D)** were not affected by sepsis CLP or Sham surgery. Further, sepsis CLP was associated with clearance of the majority of circulating leukocytes **(E)**. **(F,G)** Sepsis leads to increased plasma levels of proinflammatory cytokines. The induction of sepsis CLP tended to increase the level of TNF-α in plasma **(F)** and induced a profound rise in plasma IL-6 levels **(G)**. CLP: cecal ligation and puncture. * means *p <* 0.05, *n* = 7–8 mice per group.

To validate the induction of acute inflammation in our model of sepsis, we first measured biomarkers of inflammation. CLP-induced sepsis led to excessive loss of body weight, lower body temperature, and decreased number of circulating leukocytes. Plasma levels of circulating pro-inflammatory cytokines were increased at 24 h after induction (Figures 1A–G).

### Effects of CLP-Induced Sepsis on Perirenal White Adipose Tissue

**Figure 2:**
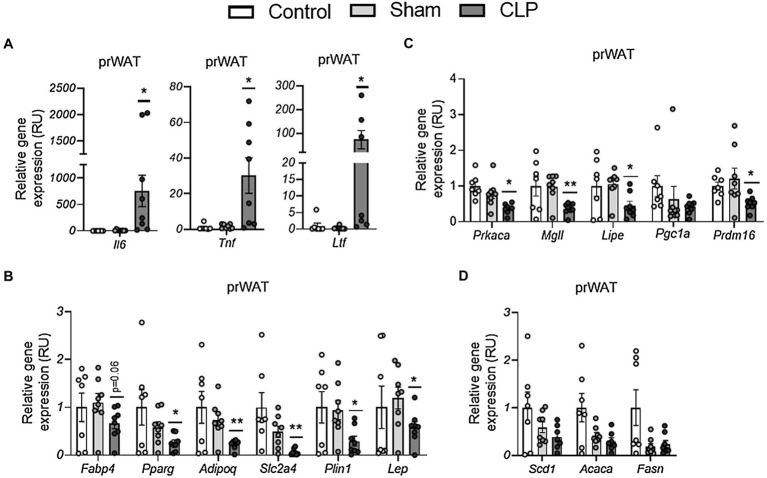
**(A–C)** The effects of CLP-induced sepsis on expression of inflammation **(A)**, adipogenesis **(B)**, lipolytic/browning **(C)**, and lipogenesis **(D)** in prWAT, . CLP: cecal ligation and puncture. ^*^p < 0.05 and ^**^p < 0.01 compared to sham, *n* = 7–8 mice per group.

In prWAT, CLP-induced sepsis resulted in increased expression of proinflammatory (*Tnf, Il6*, and *Ltf*)-related gene expression (Figure 2A) in parallel to decreased adipogenesis (*Adipoq, Pparg, Plin1, Slc2a4, Fabp4, and Lep*)-, lipolytic/browning (*Prkaca, Mgll, Lipe, and Prdm16*)-related gene expression (Figures 2B,C), but did not exert any effects on expression of *Pgc1a and* lipogenic (*Scd1, Acaca, and Fasn*)-related genes (Figures 2C,D). Thus, similar to previous studies (Tsujimura et al., 2011; Kutsukake et al., 2014), sepsis led to the induction of genes encoding pro-inflammatory cytokines in WAT, associated with decreased expression of genes related to adipogenesis. In addition, CLP-induced sepsis also attenuated catabolic pathways, reducing expression of Prdm16 and lipolysis-related genes.

### Effects of CLP-Induced Sepsis on Interscapular Brown Adipose Tissue-Related Gene Expression

**Figure 3:**
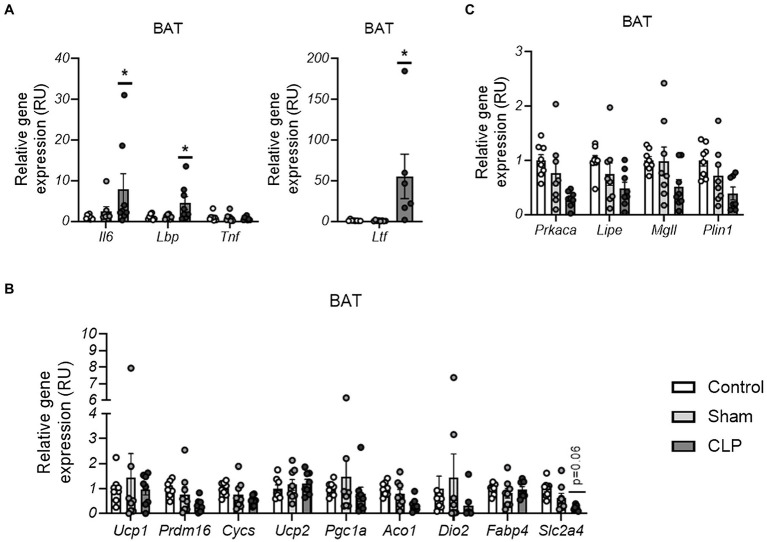
**(A–C)** The effects of CLP-induced sepsis on expression of inflammation **(A)**, thermogenesis/mitochondria **(B)**, and lipolysis **(C)**-related genes in BAT, . CLP: cecal ligation and puncture. ^*^p < 0.05 and ^**^p < 0.01 compared to sham, *n* = 7–8 mice per group.

To assess whether acute inflammation in sepsis leads to BAT inflammation and affects BAT function similar to WAT, we then measured expression of genes encoding pro-inflammatory cytokines and related to adipose tissue function in BAT. CLP-induced sepsis resulted in increased *Il6, Ltf*, and *Lbp* but not *Tnf* in BAT (Figure 3A). In contrast to WAT, no differences were found on gene expression markers of BAT activity, including thermogenic/mitochondrial (*Ucp1, Prdm16, Ucp2, Cycs, Pgc1a, Aco1, and Dio2*)- and lipolytic (*Prkaka, Mgll, Lipe, and Plin1*)-related genes, and markers of fatty acid and glucose uptake (*Fabp4 and Slc2a4*; Figures 3B,C). Thus, although sepsis also led to expression of pro-inflammatory cytokines, it was not associated with markers of adipose tissue dysfunction in BAT.

## Discussion

The current study confirms the effects of acute inflammation on adipose tissue dysfunction in perirenal (visceral) WAT, whereas in BAT, the effects of CLP-induced sepsis were less profound.

In prWAT, the main effects of CLP-induced sepsis included increased expression of proinflammatory cytokines (*Tnf* and *IL-6*) and a neutrophil marker (*Ltf*) and decreased markers of adipogenesis. The expression of *Tnf* and *IL-6* genes is widely used to characterize sepsis-induced inflammatory response in several tissues (Tsujimura et al., 2011; Chen et al., 2014; Ferlito et al., 2014; Kutsukake et al., 2014; Li et al., 2017). Even though IL-6 (Hoch et al., 2008) is expressed in adipocytes, the main biosynthesis source of IL-6 and TNF in adipose tissue is in cells of stromal vascular fraction (SVF; Almuraikhy et al., 2016). In normal conditions, *Ltf* gene was more expressed in adipocytes compared to SVF (Moreno-Navarrete et al., 2013) and an adipogenic role of *Ltf* has been demonstrated (Moreno-Navarrete et al., 2011, 2014). However, the most abundant expression of *Ltf* is found in neutrophils (Baker and Baker, 2005), which is a 100-fold higher as compared to adipose tissue (Moreno-Navarrete et al., 2013). In addition, inflammatory conditions decrease adipocyte *Ltf* mRNA (Yarmo et al., 2010) but increase neutrophil *Ltf* (Bae et al., 2014). These findings suggest that increased *Ltf* mRNA might result from enhanced neutrophil infiltration in adipose tissue in response to CLP-induced sepsis. This hypothesis is further supported by the severe decrease in the number of leukocytes in plasma in the septic mice.

Considering the negative effects of acute inflammation in adipocyte physiology and adipogenesis previously demonstrated at cellular level (Constant et al., 2006; Lacasa et al., 2007; Yarmo et al., 2010), the sepsis-induced inflammatory environment might explain the observed decreased adipogenesis.

In BAT, the rise of *LBP* mRNA was the most significant effect of CLP-induced sepsis. Interestingly, a recent study demonstrated decreased expression of *LBP* in cold-induced BAT and increased BAT activity in Lbp-null mice, suggesting that LBP was a negative regulator of the browning process (Gavaldà-Navarro et al., 2016). In the current study, increased *LBP* gene expression was not associated with a significant decrease in thermogenic or mitochondrial-related genes.

Even though *in vitro* experiments demonstrated that macrophage conditioned medium attenuated cold-induced brown adipocyte activity (Kern et al., 2014) and that the activation of receptors that mediate LPS pathway (such as TLR4, TLR2, and NOD1) inhibited UCP1 and mitochondrial respiration in brown adipocytes (Bae et al., 2014), in the present study, no significant effects of CLP-induced sepsis in expression of thermogenic and mitochondrial activity-related genes were observed.

In fact, the impact of inflammation on BAT activity is currently discussed, as some studies in mice with obesity-associated chronic low-level inflammation reported that BAT inflammation is associated with decreased thermogenic activity (Sakamoto et al., 2016; Escalona-Garrido et al., 2020), in other studies no correlation between BAT thermogenesis and inflammation was observed (Cao et al., 2018; Boulet et al., 2021). To the best of our knowledge, the *in vivo* effects of acute inflammation mediated by CLP-induced sepsis in expression of BAT thermogenic- and metabolic-related genes have not been previously reported.

Interestingly, comparing the induction of inflammation-related genes in prWAT and BAT, we found that *Tnf* gene expression was only induced in prWAT. TNFα induces insulin resistance promoting the phosphorylation of serine residues in insulin receptor substrate 1 (Hotamisligil et al., 1996). The specific increased *Tnf* gene expression in prWAT points to this cytokine as a possible mediator of WAT dysfunction through the impairment of insulin action in adipocytes. In BAT, the absence of changes in *Tnf* mRNA levels run in parallel to the absence of negative effects of CLP-induced sepsis on metabolic-related gene expression. In fact, the induction of *Tnf* mRNA in BAT enhanced lipogenic gene expression, resulting in BAT “whitening,” whereas the inhibition of this cytokine led to increased expression of lipolytic and thermogenic genes (Polyák et al., 2016).

To sum up, current data show that whereas CLP-induced sepsis promotes both WAT and BAT inflammation, this acute inflammatory condition only impacts on WAT adipogenic gene expression, without significant effects on BAT thermogenic genes, suggesting a dissociation of acute inflammation from adipose tissue dysfunction in BAT, but not WAT. However, since current study was based only on gene expression analysis, further functional experiments are required to confirm this suggestion.

## Data Availability Statement

The raw data supporting the conclusions of this article will be made available by the authors, without undue reservation.

## Ethics Statement

The animal study was reviewed and approved by the Central Committee Animal Experiments of Netherlands (protocol number 16593).

## Author Contributions

JMM-N and HB participated in study design and analysis of data, and wrote and edited the manuscript. FC, VJ, and JMF-R participated in analysis of data, and revised the manuscript critically for important intellectual content. All authors contributed to the article and approved the submitted version.

### Conflict of Interest

The authors declare that the research was conducted in the absence of any commercial or financial relationships that could be construed as a potential conflict of interest.
